# Efficacy and tolerability of Meratrim for weight management: a randomized, double-blind, placebo-controlled study in healthy overweight human subjects

**DOI:** 10.1186/s12944-016-0306-4

**Published:** 2016-08-24

**Authors:** Venkateshwarlu Kudiganti, Raveendra Ramamurthy Kodur, Sushma Raveendra Kodur, Manjunath Halemane, Dheeraj Kumar Deep

**Affiliations:** 1 Srinivasa clinic and Diabetic care center, Girinagar, BSK 3rd Stage, Bengaluru, 560 085 Karnataka India; 2 D2L Clinical Solutions, Bengaluru, 560048 India

**Keywords:** Healthy overweight, *Garcinia mangostana*, Meratrim, *Sphaeranthus indicus*, Weight management

## Abstract

**Background:**

Meratrim is a blend of two plant extracts obtained from *Sphaeranthus indicus* flower heads and *Garcinia mangostana* fruit rinds. Previous studies have demonstrated that Meratrim is effective for weight management in obese individuals. The objective of this study was to assess the efficacy and tolerability of Meratrim in managing body weight in healthy overweight subjects.

**Methods:**

Sixty participants with a mean BMI of 28.3 kg/m^2^ were randomized into two groups receiving either 400 mg of Meratrim twice daily or two identical placebo capsules for a period of 16 weeks. Subjects were asked to consume about 2,000 kcal/day throughout the study period and walk 5 days a week for 30 min daily. The primary endpoint was defined as the change in body weight from baseline to end of week 16 for the Meratrim group versus placebo. Fifty seven subjects completed the trial.

**Results:**

At study conclusion, statistically significant reductions in body weight (5.09 vs. 1.1 kg; *p* < 0.0001), BMI (1.91 vs. 0.43 kg/m^2^; *p* < 0.0001), waist (9.97 vs. 3.71 cm; *p* < 0.001) and hip size (10.38 vs. 5.11 cm; *p* < 0.0001) were observed in the Meratrim versus the placebo group. Additionally, a significant change in serum LDL (−14.79 vs. 6.25 mg/dL; *p* < 0.0001), triglycerides (−43.62 vs. -13.68 mg/dL; *p* < 0.001) and total cholesterol (−20.0 vs. -0.75 mg/dL; *p* = 0.0002) was observed in the Meratrim cohort compared to the placebo. No supplementation related adverse events were noted during the study.

**Conclusions:**

The study findings suggest that Meratrim is well-tolerated and is an effective ingredient for weight management in healthy overweight subjects.

**Trial registration:**

CTRI/2014/07/004727; www.CTRI.nic.in

## Background

The World Health Organization has acknowledged that obesity appears to be reaching epidemic proportions on a global scale [1–3]. The worldwide socioeconomic impact of this disease is estimated at approximately $2 trillion dollars, equivalent in size to the socioeconomic cost linked to cigarette smoking [4]. In light of this estimate, there continues to be a strong need for systematic interventions (e.g., education, changes in food choices and eating habits, exercise, dietary supplements, and pharmaceuticals) aimed at slowing this disease’s unabated growth.

Earlier studies reported that an herbal formulation (Meratrim) consisting of extracts of the flower heads from *Sphaeranthus indicus* and the fruit rinds of *Garcinia mangostana* demonstrated significant weight loss outcomes in two randomized, double-blind, placebo-controlled clinical studies on obese subjects [5, 6]. An 800 mg daily dose of this supplement resulted in statistically significant reductions in body weight, BMI, waist and hip circumference that exceed those achieved via diet and exercise alone. The blend’s significant effect on body weight and anthropomorphic parameters occurred as early as 2 weeks and continued to increase during the 8-week trial. Additionally, consuming the herbal blend also yielded significant improvements in lipid and glycemic serum profiles.

To evaluate the weight loss efficacy of Meratrim supplement on healthy overweight subjects, we conducted a 16-week randomized, double-blind, placebo controlled trial in healthy overweight individuals with an average BMI of 28.3 kg/m^2^. The primary objective of our study was to assess the weight loss efficacy and tolerability of Meratrim in reducing body weight. We report herein that consuming Meratrim increases weight loss that is statistically significant versus the weight loss due to diet and exercise alone, and that this ingredient is well tolerated. In addition, we also describe the possible molecular basis of anti-obesity efficacy of Meratrim in cellular models in vitro.

## Methods

### Study material

Meratrim consisted of extracts from the flower heads of *Sphaeranthus indicus* (*S. indicus*) and the fruit rinds of *Garcinia mangostana* (*G. mangostana*). The extracts were prepared using the method as previously described [5–7]. Briefly, *S. indicus* flower heads were pulverized and extracted first with methanol then with ethyl acetate to form a thick paste. Separately, *G. mangostana* fruit rinds were pulverized and extracted with 80:20 ratio of methanol to water. The solvent was removed under vacuum, and the resulting flakes were milled. The *S. indicus* paste and *G. mangostana* powdered extract were blended together in 3:1 ratio then combined with excipients (55 % w/w) to produce Meratrim. Both 7-hydroxyfrullanolide and α-mangostin served as internal standards for monitoring the batch-to-batch consistency of the *S. indicus* and *G. mangostana* extracts, respectively.

Meratrim was manufactured in a CGMP certified facility (Laila Nutraceuticals, Vijayawada, India) and encapsulated in size zero hard gelatin maroon colored capsules with the excipients microcrystalline cellulose (SANCEL-W, NB Entrepreneurs, Nagpur, India) and magnesium stearate (Magnesium stearate, Amishi Drugs and Chemicals Private Limited. Ahmedabad, India), in a batch type capsule filling equipment (MF-30, ACG PAM Pharma Technologies Pvt. Ltd, Mumbai, India). Identical placebo capsules contained only excipients were prepared in the same facility. Both Meratrim and placebo capsules were packaged in white, 100 cc HDPE screw cap bottles and submitted to Clinical Quality Assurance (QA) team. Study supplement bottles were stored at room temperature, in a secure cabinet with access limited to the clinical QA team members until distributed. Coded labels, prepared as per randomization code by QA personnel, were affixed to the study bottles. Meratrim and placebo bottles were mixed, arranged in sequential order and submitted to the study site. Study product labels conformed to all local and international clinical trial requirements and guidelines. The study site investigator, or his designate, maintained an inventory of all investigational products received, dispensed, and returned to the site by study participants during each site visit.

### Cell based studies

#### Cell culture and treatments

3T3-L1 mouse embryo fibroblasts and HepG2 human hepatocellular carcinoma cells were obtained from American Type Culture Collection (Manassas, VA) and cultivated in DMEM supplemented with 10 % fetal bovine serum (FBS) 100 U/ml penicillin, 100 μg/ml streptomycin, 1 mM sodium pyruvate and 4.5 g/L D-glucose. 3T3-L1 preadipocytes were differentiated to mature adipocytes as described previously [8]. For treatments, the dry powdered Meratrim was dissolved in DMSO and the final concentration of DMSO in the culture was 0.2 % (v/v) in all experiments. Matured adipocytes or hepatocytes were treated with desired concentration of Meratrim for various time periods; vehicle control culture wells received 0.2 % DMSO only.

#### Adipogenesis assay

Equal number of cells was plated in each well of 24-well culture plates. Cells were pre-treated with 5, 10 and 15 μg/ml of Meratrim for 2 h and followed by addition of differentiation medium containing 500 nM insulin, 1.0 μM Dexamethasone and 0.5 mM isobutylmethylxanthine (IBMX) for 48 h. Thereafter, cells were further incubated with post differentiation medium (DMEM containing 100 nM insulin) in presence or absence of different concentrations of test samples for further 8 days. The control cultures received only 0.2 % (v/v) DMSO as the vehicle. The remaining procedure was the same as described earlier [8].

#### Lipolysis assay

The intracellular lipid break down efficacy of Meratrim was evaluated by measuring the released glycerol in the 3T3-L1 culture supernatants. Briefly, equal number of 3T3-L1 preadipocytes was allowed to differentiate into mature adipocytes in each well of 24-well culture plate as stated in Adipogenesis assay method. Every culture well contained 90–95 % differentiated cells with numerous intracellular vesicles visible under microscope. Thereafter, the wells were washed two times with pre-warmed Hank’s Balanced Salt Solution (HBSS). Washed cells were treated 5, 10 and 25 μg/ml Meratrim in phenol red free DMEM supplemented with 2 % Bovine serum albumin (BSA) for 2 h. Cell culture supernatants were clarified at 10,000 g for 5 min. at 4 °C. Released glycerol content in the culture supernatants was measured following the protocol provided with Adipolysis Assay Kit (Millipore, Billerica, MA).

#### Immunoblot assay

Briefly, the treated cells were washed twice with chilled PBS and the cell lysates were prepared in a lysis buffer [8]. The cell lysates were clarified at 14,000 g for 20 min at 4 °C. The protein concentrations were estimated by Bradford reagent.

Equal amount of proteins was resolved in SDS-PAGE. Following SDS-PAGE, the electro-blotted nitrocellulose membranes were reacted with relevant primary antibody specific to AMPK, phospho-AMPK (Thr172), ACC, phospho-ACC (Ser79), Fatty Acid Synthase and Actin (Cell Signaling Technology, Boston, MA). Specific signals were detected with enhanced chemiluminescence (Thermo scientific, USA) and the signal intensities were captured in Gel Doc™ XR+ Imaging System and analyzed using Image Lab™ 2.0 Software (BioRad, Hercules, CA).

### Clinical study

#### Study design

This was a randomized, parallel group, double-blind, placebo-controlled, 16-week intervention study, performed from 30 August, 2014 to 23 February 2015, designed to assess the efficacy of Meratrim in facilitating weight loss in healthy, overweight individuals who exercise and diet. The study was performed at a single clinical center (Srinivasa Clinic & Diabetic Care Centre) in Southern India under the management of the clinical research organization D2L Clinical Solutions (Bangalore, India). The clinical trial registration number is CTRI/2014/07/004727. The study protocol was evaluated and approved by the Bangalore Ethics Committee (Bangalore, India) on 13 May 2014. The clinical center complied with all relevant ICH-GCP, the Declaration of Helsinki (1996), plus all applicable local government and institutional research policies, procedures, and guidelines. The study was conducted according to US and international standards of Good Clinical Practice (FDA Title 21 CFR part 312 and ICH guidelines).

Efficacy outcome measures, including weight, waist, and hip circumference were recorded at all visits. Lean body mass was assessed via Dual energy X-ray absorptiometry (DEXA) at randomization and at the final study visit (week 16). Both assessments were conducted after overnight fasting. Medical monitoring included a physical examination and vital signs measurements at all visits. Subject diaries and compliance cards were provided beginning at randomization through the final visit, and collected at all follow up visits. Study product was dispensed starting at the randomization visit through week 12. Study supplement bottles were collected at all follow up visits and unused capsules counted. Blood and urine sample collections were obtained at screening, weeks 4 and 8, and at study conclusion. Pregnancy testing was done at screening and all follow-up visits starting at visit 4 (week 4). Adverse events (AEs) were recorded using the subject’s diary inputs plus a site visit questionnaire administered by the study personnel at all visits.

#### Clinical endpoints

The primary endpoint for this clinical study was the reduction in body weight from baseline to the end of the treatment period. Secondary endpoints include anthropometric measurements, such as body mass index (BMI), waist and hip circumference, waist-to-hip ratio. Additional secondary endpoints include changes in serum lipid profile, increase in lean body mass and serum levels of adiponectin, leptin, ghrelin and insulin. Waist girth was measured as the smallest horizontal girth between the costal margins and the iliac crests at the end of normal expiration, while hip girth was measured at the greatest abdominal circumference at the level of greater trochanters. In addition the Visual Analog Scale (VAS) for appetite and the Profile of Mood States-Short Form (POMS-SF) were used to assess subject appetite and mood, respectively [9, 10] Both questionnaires were administered after overnight fasting. All clinical efficacy data was entered into the subjects CRF by the clinical site coordinator delegated by principal investigator (PI). CRFs were reviewed, verified and signed by the PI.

#### Subject recruitment

Sixty eligible subjects were recruited through inclusion and exclusion criteria into the study (Table 1). Healthy overweight adult men and women (21–50 years), who were willing to adhere to a vegetarian or non-vegetarian diet of approximately 2000 kcal/day consisting of 17 % protein, 25 % Fat and 58 % carbohydrate and 30 min walk for five days per week were enrolled in the study. Individuals who presented with intractable obesity, a history of chronic diseases, or personal behaviors that would confound the interpretation of results arising from this study were excluded.

**Table 1:** Inclusion and exclusion criteria

Inclusion Criteria
• Male or female subjects between 21 to 50 years of age.
• Subject with BMI range (27–32 kg/m^2^).
• Ability to understand the risks/benefits of the protocol.
• Female subjects of childbearing potential must be using a medically acceptable form of birth control. Female subjects of non-childbearing potential must be amenorrheic for at least 1 year or had a hysterectomy and/or bilateral oophorectomy.
• Willingness to participate in a walking-exercise program (30 min per day) during the course of the study.
• Subject agrees to consume a vegetarian/non-vegetarian diet of approximately 2000 kcal/day (17 % protein, 25 % Fat and 58 % carbohydrate).
• Subjects agree to come to study site in fasting condition for their weight measurement and other laboratory parameters examination in all the scheduled visits.
• Subjects should be available for the entire duration of the study (6–8 months).
• Subject using other therapies for weight management including physiotherapy/ occupational therapy agrees to discontinue these therapies during this study.
• Subject willing to go for DEXA analysis as per the scheduled visits during the study.
• Subjects agree to maintain the activity dairy.
• Subjects willing to give written informed consent and willing to comply with the trial protocol.
Exclusion Criteria
• Subjects suffered from intractable obesity, had defined weight limits or had experienced any recent, unexplained weight loss or gain.
• Subjects having history of underlying inflammatory arthropathy; septic arthritis; inflammatory joint disease; gout; pseudo-gout; Paget's disease; joint fracture; acromegaly; fibromyalgia; Wilson's disease; ochronosis; hemochromatosis; heritable arthritic disorder or collagen gene mutations or rheumatoid arthritis.
• Subjects having history of asthma, cardiovascular diseases, thyroid disease, coagulopathies, hypertension, congestive heart failure.
• Subjects having history of diabetes (Type I or Type II) - except other than the subject having the pre-diabetes condition with the fasting blood glucose between 100 to 125 mg/dl or random blood glucose ≥ 140–199 mg/dl.
• Subject with Hyperuricemia (males > 480 μmol/L, females > 450 μmol/L).
• Subjects having abnormal liver or kidney function tests (ALT or AST > 2 times the upper limit of normal; elevated Creatinine, males > 125 μmol/L, females > 110 μmol/L).
• Subjects having abnormal findings on complete blood count.
• Subjects with HIV Positive.
• Subjects having history of high alcohol intake (>2 standard drinks per day).
• Pregnant, breast feeding or planning to become pregnant during the study.
• Subjects having history of psychiatric disorder that may impair the ability of subjects to provide written informed consent.
• Any other condition that, in the opinion of the investigator, would adversely affect the subject's ability to complete the study or its measures.
• Subjects participated in any investigational drug study within thirty (30) days prior to screening.

#### Randomization and blinding

Participants were randomized 1:1 using randomization codes that were computer generated using a permuted block design consisting of 60 individuals. The randomization code was generated by an independent statistician using SPSS statistical software version 16. The randomization codes were stored centrally in opaque, sealed envelopes at the clinical site so they could be opened by medical personnel in case of a medical emergency. The randomization code was broken after the database was locked.

#### Dosing regimen

Subjects were instructed to ingest one capsule (400 mg) twice daily thirty minutes before breakfast and dinner. Dosage forms were tested using HPLC methods developed by Laila Nutraceuticals (Vijayawada, India).

#### Prior and concomitant therapies

Use of any centrally acting anorectic nutraceuticals, drugs or other agents that inhibit the absorption of nutrients or promote weight loss were prohibited two weeks prior to enrolment into the study. Enrolled subjects were not permitted to use any concomitant medications without first consulting with the study investigator. Based on the investigator’s discretion, the following OTC medications were permitted - Cetirizine, Pantoprazole, Gelusil and Nimesulide - in order to address any allergies, dyspepsia, or transient aches and pain during the course of the study.

#### Compliance and safety

Subjects were instructed to bring their study supplement bottles to each follow up visit. The number of remaining capsules in each bottle was recorded. Subjects also completed a dairy indicating daily consumption of study supplement and listing of any adverse events that might have occurred. Safety assessments were done at each clinical visit by the PI and recorded into each subject’s CRF by clinical study staff. CRFs were reviewed and signed by the PI. Adverse events were characterized according to standardized nomenclature set by MedDRA.

#### Study evaluations

Subject weights were obtained using a calibrated scale (AVON Corporation Ltd, Model No: APD802). Subject activity levels were assessed using a Digiwalker SW-701 pedometer (Yamax, San Antonio, TX). Specimens were analyzed using the Prietest clinical chemistry analyzer (ROBONIK INDIA PVT LTD, Mumbai, India). DEXA measurements were carried out using the enCORE® based X-ray Bone Densitometer; Lunar Prodigy Series (GE Medical Systems, Chakan, Pune, India). Leptin, adiponectin and insulin were quantitatively estimated in the serum samples using specific EIA based methods. Leptin (EZHL-80SK), Adiponectin (442307) and Insulin (IS130D) ELISA kits were procured from EMD Millipore Corporation (Billerica, MA), BioLegend Inc. (San Diego, CA) and Calbiotech Inc. (Spring Valley, CA), respectively. The assay procedures to estimate the biomarkers were essentially the same as recommended by the respective vendors. Briefly, the specific monoclonal antibody coated 96-well plates were reacted with the serum samples. The specific antibody-antigen bindings were probed with biotinylated detection antibody and followed by streptavidin conjugated horseradish peroxidase enzyme. Finally, the color developments were recorded in a spectrophotometer (SpectraMax M5e, Molecular Devices, Sunnyvale, CA) and the data were analyzed using SoftMax Pro v5.4. The assay sensitivities of Leptin, Adiponectin and Insulin are 0.2 ng/ml, 0.086 ng/ml and 0.4777μIU/ml, respectively.

#### Statistical analyses

All subjects randomized into the study and participated in at least one post-baseline assessment were included in all efficacy assessments (ITT). Missing observations were imputed using the last observation carried forward (LOCF) approach. Additional analyses of the primary endpoints were carried out on a per protocol basis and included completers free of protocol violations. Between group differences were determined using ANCOVA with baseline set as the covariate. The unpaired and paired t tests were used to test for between group and within group differences in those cases where the data was normally distributed. Statistical significance was set at ≤0.05. Power calculations, using previously published efficacy data [5, 6], allowed us to conclude that 20 completers per cohort was sufficient to achieve 80 % power to detect outcome differences in weight loss between the groups, assuming a two-sided significance level of 0.05. Thirty subjects per treatment group were calculated to provide more than 90 % power under identical analytical conditions. Based on these estimates, and assuming a 40 % screen failure rate, we anticipated that we would need to screen 100 individuals to obtain 60 volunteers (30 subjects per group), the targeted sample size for this study. In practice, we screened a total of 82 subjects to achieve the targeted number of randomized volunteers required. Subjects without a baseline efficacy assessment were excluded from the analysis of that efficacy variable. Safety analyses were done on an ITT basis. Statistical analyses were generated using SAS version 9.2.

## Results

### Demographics and baseline characteristics

Table 2 summarizes the demographics of the 60 subjects that were eligible for efficacy and safety analyses. The summary includes the subject’s weight, BMI, waist and hip size, as well as their blood biochemistry status. Out of 82 individuals screened, 60 subjects who met the study’s eligibility criteria were randomized to placebo or Meratrim (*n* = 30 per group).

**Table 2:** Demographic and Baseline characteristics of the subjects eligible for the trial

Characteristics	Placebo	Meratrim
	(*n* = 30)	(*n* = 30)
Sex ((n) male + (n) female)	14 M + 16 F	10 M + 20 F
Age (years)	39.47 ± 1.73	36.63 ± 1.64
Height (meters)	1.65 ± 0.017	1.63 ± 0.02
Body weight (kg)	76.56 ± 1.64	75.93 ± 1.91
Body mass index (kg/m^2^)	28.20 ± 0.24	28.48 ± 0.25
Waist circumference (cm)	103.30 ± 1.53	102.00 ± 1.59
Hip circumference (cm)	108.70 ± 1.88	108.60 ± 1.59
Waist/Hip ratio	0.96 ± 0.017	0.94 ± 0.013
Triglycerides (mg/dL)	200.50 ± 8.16	194.20 ± 8.96
Total cholesterol (mg/dL)	184.90 ± 6.18	168.80 ± 5.37
LDL (mg/dL)	108.20 ± 5.97	94.67 ± 4.73
HDL (mg/dL)	36.60 ± 0.42	35.27 ± 0.51
LHR	2.94 ± 0.15	2.68 ± 0.12
VLDL (mg/dL)	40.13 ± 1.67	38.83 ± 1.78
Fasting blood glucose (mg/dL)	99.47 ± 1.82	95.60 ± 1.99

Values represent mean ± SE

*LDL* low-density lipoproteins; *HDL* high-density lipoprotein; *LHR* LDL/HDL ratio; *VLDL* very low-density lipoproteins

### Subject dropouts

Three individuals dropped out from the study. Two were in the placebo cohort, one in the Meratrim supplementation group. The placebo group dropouts occurred at visits four and six and were due to one subject deciding to discontinue participating in the study, while the other was due to the subject’s relocation and not being able to visit the clinic. The single subject dropout for the Meratrim group occurred at visit 4 and was also due to the subject’s relocation away from the study site. The number of completers in placebo and Meratrim group was 28 and 29, respectively.

### Study compliance

Compliance with daily dosing of study capsules exceeded 95 % for all cohorts (data not shown). Readouts from the individual pedometers showed that the Meratrim and placebo groups, walked similar average distances per day during the course of this study. A detailed review of each subject diary showed that both groups complied to a high extent with the recommended diet (data not shown).

### Weight loss

By the end of the study (16 weeks), the Meratrim supplemented cohort presented an average weight loss of 5.09 kg in sharp contrast to the 1.1 kg average loss noted in the placebo group (p < 0.0001; Table 3). It is interesting to note that Meratrim supplemented group experienced gradual weight reduction from the second week of commencement of the study. At the end of the trial, Meratrim group demonstrated 6.7 % body weight reduction; in contrast, placebo recorded only 1.4 % body weight reduction (Table 3). Meratrim supplemented group experienced a net BMI reduction of 1.91±0.13 kg/m^2^ (28.46±0.25 and 26.55±0.25 kg/m^2^ at baseline and at week 16, respectively); in comparison, at the end of the study the placebo showed a reduction of BMI only 0.43±0.19 kg/m^2^. At termination, intergroup analysis between the net changes of BMI (baseline vs. termination) was significant (*p*<0.0001).

**Table 3:** Reduction in body weight in Meratrim and placebo groups over 16 week study period

Parameter Reduction	Weeks	Placebo	Meratrim	Net reduction^a,b^	*P* value
		(*n* = 28)	(*n* = 29)		
Body weight (kg)	2	0.42 ± 0.18	0.51 ± 0.16	0.09 (0.1 %)	0.749
	4	0.64 ± 0.29	1.53 ± 0.20	0.89 (1.2 %)	0.048*
	8	1.26 ± 0.40	2.10 ± 0.25	0.84 (1.1 %)	0.190
	12	1.19 ± 0.43	3.14 ± 0.28	1.95 (2.6 %)	0.002*
	16	1.10 ± 0.46	5.09 ± 0.38	3.99 (5.3 %)	<0.0001*

Values represent mean ± SE

*Significant difference between Meratrim and placebo group mean values calculated using ANCOVA with baseline as covariate

^a^Net reduction = Meratrim minus placebo

^b^Numbers in parenthesis represents the % net reduction from baseline for Meratrim group

### DEXA

At trial initiation, all study participants have undergone DEXA scan to measure body fat percentage but only 22 subjects (eleven subjects in each group) have agreed for the scan at the final evaluation. We consider that this small group size cannot be a proper representative of the overall population. This event can be classified as minor protocol deviation, as it does not influence the data constituting the primary outcome of the study, or subject well-being; and was reported to the IEC as stipulated by their bylaws and the conditions under which approval for this study was granted (data not presented).

### Changes in anthropometric parameters

Statistically significant decreases in both hip and waist size were noted as early as four weeks (Table 4). By study conclusion, the average waist size of subjects supplemented with Meratrim decreased by 9.97 cm as compared to 3.71 cm for subjects consuming placebo (*p* < 0.001). Hip size also decreased significantly (10.38 cm, *p* < 0.0001) by the end of the study. The reduction in hip size, as was noted for weight loss, underwent a significant increase in magnitude after eight weeks of supplementation. Nonetheless, the change in hip size was statistically significant versus placebo at as early as 4 weeks (3.14 cm vs. 2.21 cm, *p* < 0.035). No statistically significant change in the waist to hip ratio was noted for this study as both parameters changed similarly during the course of this research.

**Table 4:** Reduction in waist and hip circumference in Meratrim and placebo groups over 16 week study period

Parameter Reduction	Weeks	Placebo	Meratrim	Net reduction^a,b^	*P* value
		(*n* = 28)	(*n* = 29)		
Waist size (cm)	2	1.07 ± 0.34	1.45 ± 0.25	0.38 (0.4 %)	0.339
	4	2.18 ± 20.39	3.38 ± 0.37	1.20 (1.2 %)	0.016*
	8	2.82 ± 0.53	5.14 ± 0.49	2.32 (2.3 %)	0.001*
	12	3.29 ± 0.58	6.83 ± 0.58	3.54 (3.5 %)	<0.001*
	16	3.71 ± 0.70	9.97 ± 0.73	6.26 (6.2 %)	<0.001*
Hip size (cm)	2	1.04 ± 0.27	1.62 ± 0.26	0.58 (0.5 %)	0.109
	4	2.21 ± 0.37	3.14 ± 20.41	0.93 (0.9 %)	0.035*
	8	3.61 ± 0.55	4.45 ± 20.51	0.84 (0.8 %)	0.112
	12	4.32 ± 0.58	6.55 ± 0.63	2.23 (2.1 %)	0.002*
	16	5.11 ± 0.69	10.38 ± 0.85	5.27 (4.9 %)	<0.0001*

Values represent mean ± SE

*Significant difference between Meratrim and placebo group mean values calculated using ANCOVA with baseline as covariate

^a^Net reduction = Meratrim minus placebo

^b^Numbers in parenthesis represents the % net reduction from baseline for Meratrim group

### Change in biochemistry biomarkers

Consistent with the previous observations [5], the present study demonstrated that Meratrim supplementation significantly reduced the serum levels of triglycerides (−43.6 mg/dL vs −13.7 mg/dL; *p* < 0.001) and total cholesterol (−20.0 mg/dL vs −0.8 mg/dL; *p* < 0.002) over the reductions obtained by diet and exercise alone (Table 5). Significant reductions in serum levels of LDL (*p* < 0.0001) and VLDL (*p* = 0.011) were also observed for the Meratrim supplemented cohort over placebo. Interestingly, at the end of the study, Meratrim provided an increase in serum HDL level by 2.03±0.75 mg/dL, whereas, in placebo HDL level was reduced by 1.96±0.48mg/dL, and this improvement is statistically significant (*p* < 0.0001) (Table 5). Changes in fasting glucose levels in both the groups from the baseline were not significant (Table 5).

**Table 5:** Changes in biochemical parameters after 16 weeks of supplementation

Parameter	Placebo	Meratrim	*P* value
	(*n* = 28)	(*n* = 29)	
Liver function			
SGOT (U/L)	−1.89 ± 0.83	−1.66 ± 0.97	0.2999
SGPT (U/L)	−2.22 ± 0.85	−3.65 ± 0.94	0.0181
Renal function			
BUN (mg/dL)	−1.48 ± 0.26	−1.65 ± 0.29	0.1938
Creatinine (mg/dL)	−0.15 ± 0.03	−0.15 ± 0.03	0.7998
Metabolic panel			
Triglycerides (mg/dL)	−13.68 ± 5.17	−43.62 ± 6.74	<0.001*
Cholesterol (mg/dL)	−0.75 ± 5.10	−20.00 ± 2.88	0.0002*
LDL (mg/dL)	6.25 ± 4.79	−14.79 ± 2.93	<0.0001*
HDL (mg/dL)	-1.96±0.48	2.03±0.75	<0.0001*
LHR	0.35 ± 0.14	−0.49 ± 0.11	<0.0001*
VLDL (mg/dL)	−5.04 ± 1.16	−7.24 ± 1.08	0.011*
Fasting glucose (mg/dL)	−10.64 ± 2.05	−9.41 ± 1.13	0.8926

Values represent mean ± SE

*BUN* Blood Urea Nitrogen, *SGOT* Serum Glutamic Oxaloacetic Transaminase; *SGPT* Serum Glutamic-Pyruvic Transaminase; *LDL* low-density lipoproteins; *HDL* high-density lipoprotein; *LHR* LDL/HDL ratio; *VLDL* very low-density lipoproteins

*Significant difference between Meratrim and placebo group mean values calculated using ANCOVA with baseline as covariate

### VAS-appetite & POMS-short form questionnaires

At study conclusion, the mean score of Visual Analog Scale (VAS) for appetite in placebo was increased by 207.4 units, while the score in Meratrim supplemented group was increased by only 23.6 units (Table 6). The intergroup analyses on the difference of VAS-appetite score at the end of the study reveal that Meratrim supplemented group experienced a significantly (*p* < 0.001) lower VAS-appetite score, in comparison with the placebo (Table 6).

**Table 6:** Mood and appetite scores after 16 weeks of supplementation

Parameter	Week	Placebo	Meratrim	*P* value
		(*n* = 28)	(*n* = 29)	
Total Mood Disturbance (TMD)	0	84.29 ± 8.210	74.93 ± 10.93	<0.001*
	16	72.89 ± 10.31	39.76 ± 15.54	
Total VAS Score	0	170.4 ± 28.58	229.8 ± 51.31	<0.001*
	16	377.8 ± 58.01	253.4 ± 45.46	

Values represent mean ± SE

*Significant difference between Meratrim and placebo group calculated using ANCOVA with baseline as covariate

A statistically significant reduction in the mean total mood disturbance (TMD) score was observed in subjects supplemented with Meratrim over placebo (72.89 versus 39.76; *p* < 0.0001). This decline in TMD is significant after 16 weeks of Meratrim supplementation (Table 6). Compared to baseline, the reduction in TMD scores was 46.93 % and 13.52 % for Meratrim and placebo group, respectively.

### Serum biomarkers

At study conclusion we did not obtain any statistically significant changes in adiponectin, leptin, ghrelin or insulin levels (Table 7). Despite the lack of statistical significance, we did note some interesting trends. In the case of adiponectin, Meratrim consumption resulted in an average increase of 398.2 ng/ml while the placebo group presented with a mean reduction of 1,030.2 ng/mL. These changes are consistent with our previous observations [5]. Serum leptin and ghrelin levels declined similarly for both the Meratrim and the placebo groups over their respective baseline levels, an effect that may be due to improved diet and to increased exercise. At study conclusion, insulin levels increased similarly for both groups.

**Table 7:** Changes in serum biomarkers after 16 weeks of supplementation

Parameter	Placebo	Meratrim	*P* value
	(*n* = 28)	(*n* = 29)	
Adiponectin (ng/mL)	−1030.2 ± 884.8	398.2 ± 1396.6	0.3940
Leptin (ng/mL)	−15.49 ± 2.83	−18.32 ± 2.56	0.4620
Ghrelin (pg/mL)	−35.92 ± 29.22	−39.79 ± 19.92	0.9138
Insulin (μIU/mL)	5.65 ± 2.31	8.44 ± 2.66	0.4336

Values represent mean ± SE

*p* values from unpaired *t* test between groups and variable sample size

### In vitro anti-adipogenic efficacy

**Fig. 1:**
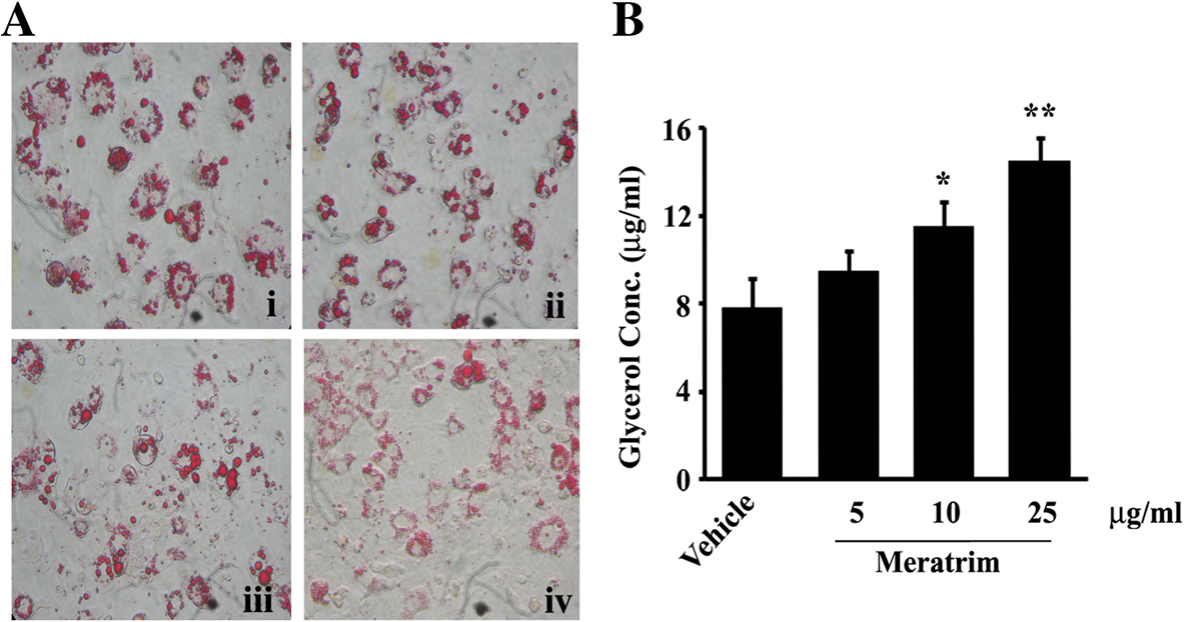
**a** Meratrim reduces lipid accumulation in 3T3-L1 adipopcytes. 3T3-L1 cells were allowed to differentiate in absence or presence of different concentrations of Meratrim, and the cells were stained with Oil Red O. Photomicrographs show intracellular lipid staining in vehicle (0.2 % DMSO), and 5, 10, 15 μg/ml Meratrim treated cells in i, ii, iii and iv, respectively. **b** Meratrim enhances lipolysis in 3T3-L1 adipocytes. Differentiated 3T3-L1 adipocytes were incubated with 5, 10, 25 μg/ml Meratrim for 2 h. Vehicle treated control cultures received only 0.2 % DMSO. Each bar represents mean (μg/ml) ± SD of released glycerol. *n* = 5. * *p* < 0.05, ** *p* < 0.005 vs. vehicle

Anti-adipogenic efficacy of Meratrim was investigated in 3T3-L1 adipocytes at in vitro conditions. Mouse pre-adipocyte 3T3-L1 cells were differentiated in presence or absence of 5, 10 and 15 μg/ml Meratrim as indicated. Oil Red O lipid staining revealed that Meratrim reduced intracellular lipid accumulation in a dose-dependent manner (Fig. 1a). Quantitative analyses indicated that in comparison with the vehicle (0.2 % DMSO) treated cultures, Meratrim treatment exhibited 16.34 % ± 3.09 (*p* = 0.0195), 25.76 % ± 4.18 (*p* = 0.0071) and 41.77 % ± 6.76 (p = 0.0001) inhibition in adipogenesis at 5, 10, 15 μg/mL, respectively (data not shown).

In addition, Meratrim exhibited a dose dependent increase of intracellular lipolytic efficacy (measured as glycerol secretion) in matured 3T3-L1 adipocytes (Fig. 1b). Mature adipocytes treated with 5, 10, 25 μg/mL Meratrim produced 9.54 ± 0.96, 11.49 ± 1.12 and 14.52 ± 1.02 μg/mL glycerol, respectively; whereas, glycerol content in the control culture medium was 7.83 ± 1.35 μg/mL. Therefore, Meratrim dose-dependently increased glycerol production in adipocyte cultures (in comparison with control) by 20.76 % (*p* = 0.0623), 46.75 % (*p* = 0.0091) and 85.43 % (*p* = 0.0018), respectively.

### Meratrim down-regulates fatty acid synthase

**Fig. 2:**
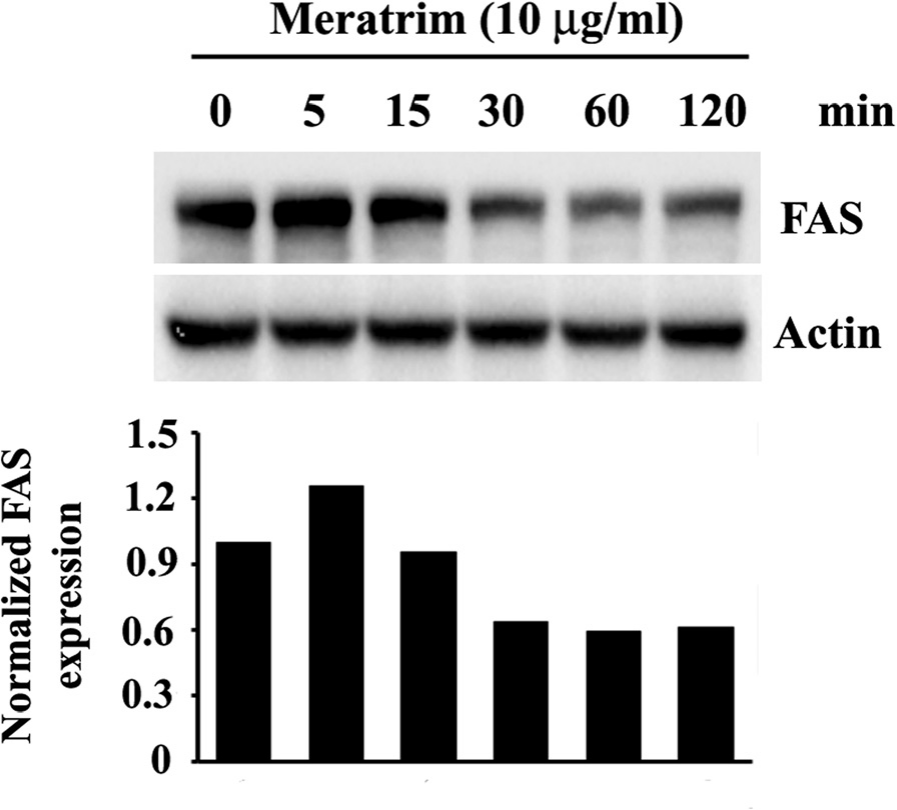
Meratrim down regulates Fatty acid synthase (FAS) protein expression in 3T3-L1 adipocytes. 3T3-L1 mature adipocytes were treated with 10 μg/ml Meratrim for indicated time periods. Representative immuno blots depict protein expression of FAS in respective treatments as indicated, actin protein was evaluated as the internal control. Each protein expression was measured densitometrically and normalized with actin expression. The normalized expressions FAS protein is represented as bar diagram

Fatty acid synthase (FAS) is the key enzyme for de novo lipogenesis in liver and fat cells [11]. Immunoblot experiments demonstrate that Meratrim treatment reduced FAS protein expression in 3T3-L1 adipocytes. Densitometric analyses on normalized FAS protein expression reveal that Meratrim down regulates the intracellular enzyme expression by 34.3 % in 30 min (Fig. 2).

### Meratrim up-regulates AMP Activated Protein Kinase (AMPK) and Acetyl CoA Carboxylase (ACC) Phosphorylation

**Fig. 3:**
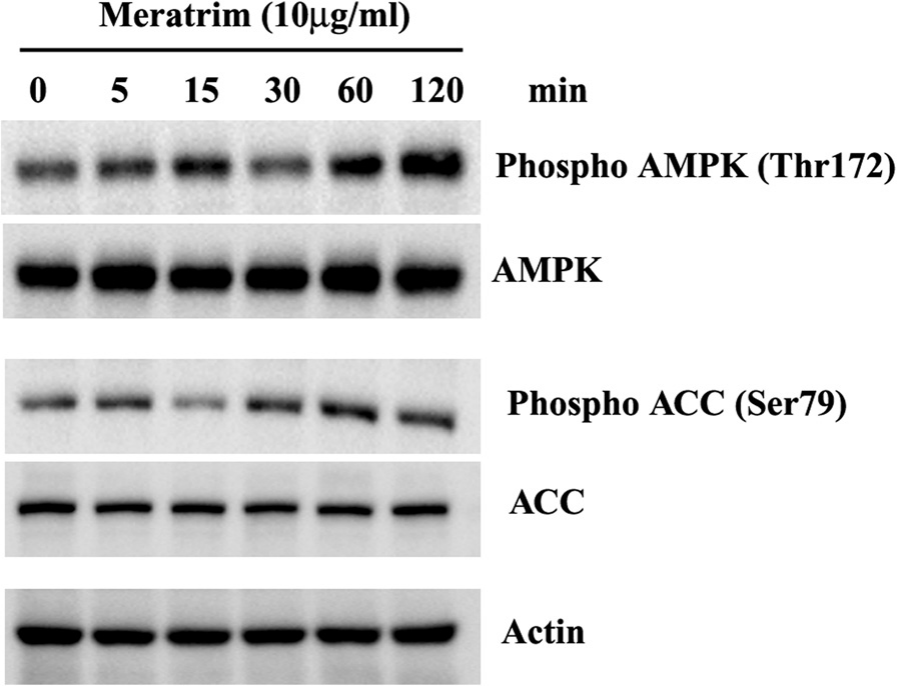
Meratrim up regulates AMPK phosphorylation in HepG2 human hepatocytes. HepG2 cells were treated with 10 μg/ml Meratrim for indicated time periods, Protein expressions of AMPK/phospho-AMPK^Thr172^ and ACC/phospho-ACC^Ser79^ have been estimated in cell lysate proteins using immunoblot assay. Expression of actin protein was evaluated as the internal control

To examine the effect of Meratrim in hepatocytes, we treated the HepG2 cells with Meratrim at different time periods as indicated. Immunoblot experiments reveal that Meratrim phosphorylates AMPK at Thr172 in HepG2 human hepatocytes, with no change in protein level detected using antibody specific to the un-phosphorylated protein. Subsequently, immunoblot experiments also revealed that ACC, the target protein of AMPK has also phosphorylated at Ser79 (Fig. 3). Together, these observations confirm that Meratrim activates AMPK in HepG2 hepatocytes.

### Safety assessments

A statistically significant decrease in SGPT levels were noted in the Meratrim cohort versus the placebo. As this change was within the physiologically normal range for SGPT, we anticipate that there is no clinical relevance. No additional differences were seen in the liver or the kidney function panel for both the study groups (Table 5).

No serious adverse events were reported in this trial (Table 8). Nine minor adverse events occurred in the placebo group, while seven were confined to the Meratrim group. These minor adverse events in the Meratrim group included dyspepsia, acidity, nausea, and gastritis. It was the PI’s medical opinion that none of these adverse events were related to consumption of study supplement. The adverse events reported for the placebo group include skin rash, itching, headache, giddiness, gastritis and dyspepsia, and feet swelling.

**Table 8:** Summary of analysis of adverse events (AEs) in all subjects

	Study group	
	Placebo (*n* = 30)	Meratrim (*n* = 30)
Severity		
Mild	9	7
Moderate	0	0
Severe	0	0
Relationship to Test Article		
Not related	9	7
Possible	0	0
Definite	0	0
Body System and AEs		
Gastrointestinal		
Acidity	0	2
Dyspepsia	2	3
Nausea	1	1
Gastritis	1	1
Pain		
Headache	1	0
Dermatology		
Itching	1	0
Rash on forearm	1	0
Neurology		
Giddiness	1	0
Circulatory		
Feet swelling	1	0
Total Number of Adverse Events Experienced During Study	9	7
Total Number of Subjects Experiencing Adverse Events: n (%)	6/30 (20 %)	5/30 (17 %)

## Discussion

We assessed the ability of Meratrim to manage weight loss in healthy overweight subjects whose average BMI was 28.3 kg/m^2^. The results presented herein demonstrate that individuals consuming Meratrim experienced greater weight loss and reductions in waist and hip size compared to individuals consuming placebo. The Meratrim supplemented group got benefit by losing an average body weight of 5.09 kg at the end of the study, which equals to 6.7 % of their mean body weight at the baseline. As all participants in this study were counseled on diet control and exercise, and the subject diaries and pedometer data showed similar levels of compliance, we conclude that the weight loss attributable to Meratrim consumption is incremental to the weight loss obtained via diet and exercise alone.

In this study we tested whether Meratrim consumption by overweight individuals might impact their blood glucose and lipid levels similar to what we previously reported [5]. We found that Meratrim reduced blood levels of triglyceride and total cholesterol in a statistically significant and a clinically relevant manner. However, unlike our previous results we did not observe an impact on blood glucose levels despite an increase in adiponectin levels, which has been shown to inversely correlate with reductions in blood glucose levels [13, 14]. This was not a surprising outcome. In prior work, the obese population we employed presented with fasting blood glucose levels that ranged from 107 mg/dL to 109 mg/dL, clearly above the normal range for this marker and indicative of prediabetes. Meratrim supplementation reduced fasting glucose levels from 109 mg/dL to 96 mg/dL after usage for two months [13, 14]. In the present study, the fasting glucose levels were only in the high end of the normal range and declined similarly for both cohorts by study conclusion. In our previous work, Meratrim supplementation also led to a statistically significant improvement in serum adiponectin levels [5]. However, while we did observe an increase in adiponectin levels, it was not significantly different when compared with the placebo group. We strongly believe that these differences in outcome are due to tangible, biochemical differences that exist between the previously studied population, and the one we report on herein [12]. These differences may also account for the lack of statistically significant changes in ghrelin and leptin levels. Although, the improvements are not statistically different, the trends suggest that Meratrim helps to control hunger and satiety signaling. Interestingly, we also observed that Meratrim supplementation increased serum insulin level by 2.7μIU/ml (over placebo). This indicates that Meratrim might increase beta cell function and helps in lowering the risk of diabetes.

In line with the significant reduction in body weight noted for the Meratrim cohort, we also observed a statistically significant change in this group’s waist and hip sizes. By study conclusion, the net reductions in hip and waist size were 4.9 % and 6.2 % of baseline values, respectively. However, these changes did not impact the waist-to-hip ratio sufficiently at study conclusion to achieve statistical significance.

Both emotional well-being and psychosocial functioning are greatly affected by obesity, and weight reduction has been shown to improve both of these mental states [15–19]. Consistent with these findings, we found that quality of life parameters, as measured by the Profile of Moods Short Form, improved significantly (47 %) for subjects consuming the herbal supplement. Interestingly, at the end of the study the VAS appetite level in Meratrim group was significantly lowered in comparison with the placebo group. These observations are strongly in line with the leptin and ghrelin levels in serum, which showed they were lower for the Meratrim cohort as compared to the placebo group. These observations together strengthen the fact that Meratrim improves emotional well-being and provides better control in hunger/satiety balance in healthy overweight individuals.

At study conclusion we were unable to obtain sufficient DEXA data to determine whether the ingestion of Meratrim resulted in a loss of lean or fat body mass. This protocol deviation was deemed as minor as this was not a primary outcome of this study. It was reported to the IEC as required by their bylaws. Root cause analyses determined that some subjects during baseline evaluation experienced DEXA as a tedious process and were reluctant to undergo the same process at 16 week DEXA scan.

In vitro experiments in cellular models demonstrate that Meratrim inhibits adipogenic differentiation and boosts lipid breakdown in mature fat cells. Earlier studies demonstrate that nutrients and hormones control lipogenesis in adipocytes through directly regulating the key enzymes of the lipogenic pathway [20, 21]. Acetyl CoA Carboxylase (ACC) and Fatty acid synthase (FAS) are the crucial enzymes in de novo lipogenesis, catalyzing the sequential enzymatic steps in the conversion of acetyl-CoA to malonyl-CoA to palmitate, respectively. After a series of reactions, palmitate is further converted into complex fatty acids. However, Fatty acid synthase (FAS) is the key rate-limiting enzyme for de novo lipogenesis [22]. Our experiments demonstrate that Meratrim has significant potential to down regulate FAS synthesis and to phosphorylate ACC at Ser79. ACC is deactivated upon its phosphorylation at Ser79 by activated AMPK (phosphorylated at Thr172) [23, 24]. Interestingly, immunoblot experiment demonstrates that Meratrim phosphorylates AMPK at Thr172 in hepatocytes; this clearly indicates that Meratrim activates AMPK. AMPK activation is associated with the stimulation of fatty acid oxidation, glucose uptake by muscle cells [25–28], inhibition of cholesterol and triglyceride synthesis [29]. AMPK activating potential of Meratrim might be the basis of significant reduction in serum triglyceride and cholesterol of the active group in the present study. In summary, our observations together strongly suggest that Meratrim inhibits de novo lipogenesis process and increases fat burning through beta oxidation. These evidences provide strong support in favor of the possible molecular mechanism underlying the anti-obesity effect of Meratrim. In addition, our observations also suggest that Meratrim helps in maintaining healthy lipid profile in overweight individuals.

## Conclusion

 We previously showed that Meratrim did not impact any clinical chemistry biomarkers which assessed the physiological health and functionality of the liver, the kidneys, the heart, or blood. These observations have been confirmed in the current trial. These results add to the growing body of evidence which suggests that Meratrim is well tolerated and has a broad-spectrum safety based on a battery of toxicological studies. In conclusion, the results presented herein validate that Meratrim is a well-tolerated and effective ingredient for weight management in healthy overweight individuals over a 16-week study period.

## References

[1] Flegal KM, Carroll MD, Ogden CL, Curtin LR: Prevalence and Trends in Obesity a`mong US Adults 1999–2008. JAMA 2010, 235–241 10.1001/jama.2009.2014 20071471

[2] Ng M, Fleming T, Robinson M, Thomson B, Graetz N, Margono C (2014). Global, regional, and national prevalence of overweight and obesity in children and adults during 1980–2013: a systematic analysis for the Global Burden of Disease Study 2013. Lancet.

[3] Schiller JS, Lucas JW, Ward BW, Peregoy JA (2010). Summary health statistics for U.S. adults: National Health Interview Survey. Vital Health Stat.

[4] Dobbs R, Sawers C, Thompson F, Manyika J, Woetzel J, Child P, McKenna S, Spatharou A: Overcoming obesity: An initial economic analysis. 2014, McKinsey & Company: www.mckinsey.com/mgi. p. 1–106.

[5] Stern JS, Peerson J, Mishra AT, Sadasiva Rao MV, Rajeswari KP (2013). Efficacy and tolerability of an herbal formulation for weight management. J Med Food.

[6] Stern JS, Peerson J, Mishra AT, Sadasiva Rao MV, Rajeswari KP (2013). Efficacy and tolerability of a novel herbal formulation for weight management. Obesity (Silver Spring).

[7] Saiyed ZM, Sengupta K, Krishnaraju AV, Trimurtulu G, Lau FC, Lugo JP (2015). Safety and toxicological evaluation of Meratrim: an herbal formulation for weight management. Food Chem Toxicol.

[8] Sengupta K, Golakoti T, Chirravuri VR, Marasetti AK (2011). An herbal formula LI85008F inhibits lipogenesis in 3T3-L1 adipocytes. Food Nutr Sci.

[9] Shacham S (1983). A shortened version of the Profile of Mood States. J Pers Assess.

[10] Flint A, Raben A, Blundell JE, Astrup A (2000). Reproducibility, power and validity of visual analogue scales in assessment of appetite sensations in single test meal studies. Int J Obes Relat Metab Disord.

[11] Hellerstein MK (1999). De novo lipogenesis in humans: metabolic and regulatory aspects. Eur J Clin Nutr.

[12] Vice E, Privette JD, Hickner RC, Barakat HA (2005). Ketone body metabolism in lean and obese women. Metabolism.

[13] Yamauchi T, Kamon J, Minokoshi Y, Ito Y, Waki H, Uchida S, Yamashita S (2002). Adiponectin stimulates glucose utilization and fatty-acid oxidation by activating AMP-activated protein kinase. Nat Med.

[14] Yamauchi T, Kamon J, Waki H, Terauchi Y, Kubota N, Hara K (2001). The fat-derived hormone adiponectin reverses insulin resistance associated with both lipoatrophy and obesity. Nat Med.

[15] Riazi A, Shakoor S, Dundas I, Eiser C, McKenzie SA (2010). Health-related quality of life in a clinical sample of obese children and adolescents. Health Qual Life Outcomes.

[16] Swallen KC, Reither EN, Haas SA, Meier AM (2005). Overweight, obesity, and health-related quality of life among adolescents: the National Longitudinal Study of Adolescent Health. Pediatrics.

[17] Kawachi I (1999). Physical and psychological consequences of weight gain. J Clin Psychiatry.

[18] Loux TJ, Haricharan RN, Clements RH, Kolotkin RL, Bledsoe SE, Haynes B, Leath T, Harmon CM (2008). Health-related quality of life before and after bariatric surgery in adolescents. J Pediatr Surg.

[19] Raben A, Tagliabue A, Astrup A (1995). The reproducibility of subjective appetite scores. Br J Nutr.

[20] Jones BH, Standridge MK, Moustaid N (1997). Angiotensin II increases lipogenesis in 3T3–L1 and human adipose cells. Endocrinology.

[21] Claycombe KJ, Jones BH, Standridge MK, Guo Y, Chun JT, Taylor JW, Moustaid-Moussa N (1998). Insulin increases fatty acid synthase gene transcription in human adipocytes. Am J Physiol.

[22] Ameer F, Scandiuzzi L, Hasnaina S, Kalbacher H, Zaidia N (2014). De novo lipogenesis in health and disease. Metabolism.

[23] Hardie DG, Carling D (1997). The AMP activated protein kinase: fuel gauge of the mammalian cell?. Eur J Biochem.

[24] Witters LA, Kemp BE (1992). Insulin activation of acetyl-CoA carboxylase accompanied by inhibition of the 5′-AMP-activated protein kinase. J Biol Chem.

[25] Winder WW, Hardie DG (1999). AMP-activated protein kinase, a metabolic master switch: possible roles in type 2 diabetes. Am J Physiol.

[26] Hayashi T, Hirshman MF, Kurth EJ, Winder WW, Goodyear LJ (1998). Evidence for 5′AMP-activated protein kinase mediation of the effect of muscle contraction on glucose transport. Diabetes.

[27] Merrill GF, Kurth EJ, Hardie DG, Winder WW (1997). AICA riboside increases AMP-activated protein kinase, fatty acid oxidation, and glucose uptake in rat muscle. Am Physiol.

[28] Goodyear LJ (2000). AMP-activated protein kinase: a critical signaling intermediary for exercise- stimulated glucose transport?. Exer Sport Sci Rev.

[29] Hardie DG (2004). AMP-activated protein kinase: a master switch in glucose and lipid metabolism. Rev Endocr Metab Disord.

